# Large-scale phenomics analysis of a T-DNA tagged mutant population

**DOI:** 10.1093/gigascience/gix055

**Published:** 2017-07-13

**Authors:** Hshin-Ping Wu, Fu-Jin Wei, Cheng-Chieh Wu, Shuen-Fang Lo, Liang-Jwu Chen, Ming-Jen Fan, Shu Chen, Ien-Chie Wen, Su-May Yu, Tuan-Hua David Ho, Ming-Hsin Lai, Yue-ie C. Hsing

**Affiliations:** 1 Institute of Plant and Microbial Biology, Academia Sinica, 128, Section 2, Yien-chu-yuan Road, Nankang, Taipei 115, Taiwan; 2 Institute of Plant Biology, National Taiwan University, 1, Section 4, Roosevelt Road, Taipei 106, Taiwan; 3 Institute of Molecular Biology, Academia Sinica, 128, Section 2, Yien-chu-yuan Road, Nankang, Taipei 115, Taiwan; 4 Agricultural Biotechnology Center, National Chung Hsing University, 145, Xingda Road, Taichung 402, Taiwan; 5 Institute of Molecular Biology, National Chung Hsing University, 145, Xingda Road, Taichung 402, Taiwan; 6 Department of Biotechnology, Asia University, 500, Lioufeng Road, Taichung 413, Taiwan; 7 Plant Germplasm Division, Taiwan Agricultural Research Institute, 189, Zhongzheng Road, Taichung 413, Taiwan; 8 Department of Life Sciences, National Chung Hsing University, 145, Xingda Road, Taichung 402, Taiwan; 9 Crop Science Division, Taiwan Agricultural Research Institute, 189, Zhongzheng Road, Taichung 413, Taiwan; 10 Department of Agronomy, National Taiwan University, 1, Section 4, Roosevelt Road, Taipei 106, Taiwan

**Keywords:** flanking sequence, large-scale phenomics, rice, sequence analysis, T-DNA insertional mutants

## Abstract

Rice, *Oryza sativa* L., is one of the most important crops in the world. With the rising world population, feeding people in a more sustainable and environmentally friendly way becomes increasingly important. Therefore, the rice research community needs to share resources to better understand the functions of rice genes that are the foundation for future agricultural biotechnology development, and one way to achieve this goal is via the extensive study of insertional mutants. We have constructed a large rice insertional mutant population in a *japonica* rice variety, Tainung 67. The collection contains about 93_ _000 mutant lines, among them 85% with phenomics data and 65% with flanking sequence data. We screened the phenotypes of 12 individual plants for each line grown under field conditions according to 68 subcategories and 3 quantitative traits. Both phenotypes and integration sites are searchable in the Taiwan Rice Insertional Mutants Database. Detailed analyses of phenomics data, T-DNA flanking sequences, and whole-genome sequencing data for rice insertional mutants can lead to the discovery of novel genes. In addition, studies of mutant phenotypes can reveal relationships among varieties, cultivation locations, and cropping seasons.

## Mutation Resource Description

### Purpose of data acquisition

With the rising world population, feeding people in a more sustainable and environmentally friendly way becomes increasingly important. Toward this end, the rice research community needs to share resources to better understand functions of rice genes and their roles in phenotypes, especially genes encoding important agronomic traits. Large-scale analyses of the relationship between sequence changes and mutant phenotypes in both forward and reverse directions have been used extensively in animal and plant research in order to investigate gene functions. An important way to define the function of a rice novel gene is to abolish or activate its expression by using a tagged sequence such as T-DNA [e.g., 1, 2], *Tos17* [3]*, Ac/Ds* [e.g., 4], or *Spm* [e.g., 5] using different rice varieties. Many research groups have established rice insertional mutant resources and provided flanking sequence tag (FST) information for these mutant lines. As of October 2016, about 450 000 integration sites were available in public databases, such as RiceGE [6], OryGenesDB (OryGenesDB, RRID:SCR_013226) [7], RAPdb [8], and NCBI Genome Survey Sequences (GSS) [9]. Several recent papers [10–12] reviewed these rice mutant resources and their applications. Nipponbare, a photoperiod-sensitive variety, was used for at least half of these resources, and the current available FST information is approaching the estimated saturation level.

However, much less effort has been devoted to phenomics analyses for these mutant lines. For instance, there are 27_ _832 phenotype records for the *Oryza* Tag Line (OTL) resource in France [13], 50_ _000 for the *Tos17* resource in Japan [14], 31_ _000 for the Rice Mutant Database in China [15], and 78_ _769 for the Taiwan Rice Insertional Mutants (TRIM) database in Taiwan [2, 16]. Thus, the total number with phenomics information is less than half of the FST data.

To establish a large-scale resource for studying rice gene functions, we used a local photoperiod-insensitive variety, constructed vectors with both knock-out and activation functions, and continued to generate mutant lines over a decade. Breeders also joined our team to provide detailed phenomics information. The FST information, a user-friendly genome browser, and the phenomics data are all available online. In addition, all seeds were stored in high-quality facilities, and T_2_ seeds are available upon request. Thus, we provide a valuable resource for rice gene functional genomics studies.

### Methods

Using T-DNA as a tagged sequence with a local *japonica* rice variety, Tainung 67 (TNG67), we prepared a large rice insertional mutant resource in Taiwan—TRIM [1, 2, 16]. The T-DNA sequence we used provided 3 functions: gene knock-out, gene activation, and promoter trapping. We started the work in 2002 and have accumulated many mutant lines as well as phenomics and flanking sequence data. All of these data are searchable at the TRIM website [17]. With an application to the T-DNA Tagged Rice Service Center [18], researchers can receive 30 T_2_ seeds for each line requested.

Many TRIM lines have been used for several forward and reverse genetics analyses [reviewed in 2]. For instance, we studied the relationship between flanking sequences and phenomics data by offspring segregation, gene expression, and overexpression and confirmed 3 genes controlling the large-grain trait and seed yield [2]. Thus, detailed data mining of both flanking sequence and phenomics data may provide useful information for investigating important agronomic traits. Here we report the current status of this valuable genetic resource and discuss an efficient way to use it, as well as differences among 3 rice phenomics populations.

## Phenomics Data Description and Analysis

### Breeders perform phenotyping in an experimental field for genetically modified plants

Since the *japonica* rice variety TNG67 is not sensitive to day length and temperature, it can be grown in 2 cropping seasons each year. The total rice growing time in Taiwan each year is about 10 months, with 2 cropping seasons of about 4 to 5 months each. The TRIM line numbers are assigned according to the chronologic order of their generation. The smallest number is M0000031, and the highest is M0127550. Our seed collection contains 86_ _310 lines with T_1_ seeds (collected from the T_0_ plants) and 78_ _757 lines with T_2_ seeds. While we propagated the T_2_ seeds, we performed phenomics screening of T_1_ plants grown in the field (Nungliang #0961050799, issued 3 August 2007), which has been carried out since the second cropping season in 2002 (Table S1). These T_1_ plants were grown in the field in a single seed–descent manner.

In total, 68 traits, belonging to 11 categories (Table S2), were screened by 5 well-trained breeders. In addition, 3 quantitative traits were recorded for each line, including plant height, panicle number, and heading date. Six seed traits were screened for about 10_ _000 lines; these include germination rate, seed length, seed width, seed height, 100-seed weight, and seed length/width ratio. Detailed methods for phenomics studies were published in a protocol-type review [19].

**Figure 1:**
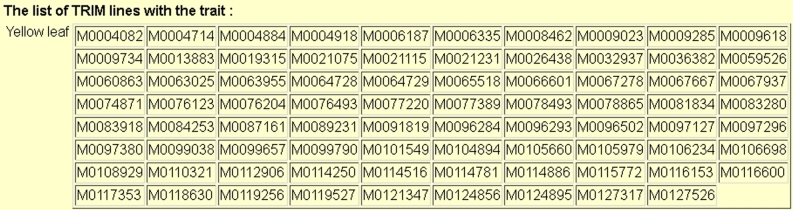
Example of search results for the trait “yellow leaf” in the TRIM resource.

Altogether, 92_ _644 lines have been grown; 62_ _375 did not have any detectable mutant traits during growth under normal field conditions. For lines with clearly visible mutant phenotypes, 14_ _129, 1993, 244, 26, and 2 lines contained 1, 2, 3, 4, and 5 groups of mutant traits, respectively; that is, 17.7% of the T_1_ population contained mutated traits. In our previous paper on phenomics [16], the mutation percentage was estimated at 17.9% in a 22_ _665 T_1_ population. Thus, the mutation frequency remained similar over the decade. The 68 traits of all TRIM lines can be searched online at the TRIM website (Fig. 1). The phenomics data for all TRIM lines are available in Table S3.

**Figure 2:**
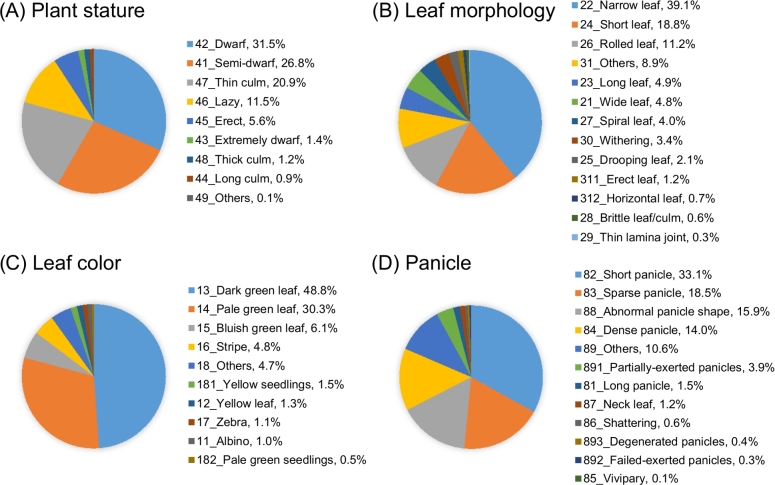
Trait percentages for the 4 high abundant phenotype categories in the TRIM resource: **(A)** plant stature; **(B)** leaf morphology; **(C)** leaf color; and **(D)** panicle.

Among the lines screened, the most frequently occurring phenotype categories are plant stature and leaf morphology (Table 1). Together, about half of the mutant traits belong to these 2 categories. The least-occurring categories are heading date, development, and lesion mimic: less than 5% of the mutant traits belong to these 3 categories. Fig. 2 shows the 4 most frequent phenotype categories. The plant stature category contains 9 traits (Fig. 2A); among them, dwarf, thin culm, and lazy canopy categories constitute more than 90% of mutants in this category. Leaf morphology has 13 traits (Fig. 2B), with the most frequent being narrow, short, rolled, long, and wide leaf. Leaf color has 10 traits (Fig. 2C), with the most frequent being dark-green, pale-green, bluish-green, and striped leaf. Notably, dark-green leaf represents about half of the traits. Panicle mutation has 12 traits (Fig. 2D), with the most frequent being short, sparse, and dense panicle, and neck leaf. Table S3 lists the phenomics records for all the TRIM lines we have observed to date. These are also searchable at the TRIM website using the phenotype trait, as shown in Fig. 1.

**Table 1:** Frequency of phenotypes in the Taiwan Rice Insertional Mutant (TRIM) library

Categories	Lines	Percentage
Plant stature	11_ _059	24.51
Leaf morphology	9107	20.18
Leaf color	5764	12.77
Fertility	5170	11.46
Panicle	5144	11.40
Grain	2862	6.34
Glume	2433	5.39
Tiller position	1635	3.62
Heading date	719	1.59
Lesion mimic	643	1.42
Development	590	1.31
Total number of lines	45_ _126	45_ _126
Percentage		100.00

## Flanking Sequence Data Description

**Figure 3:**
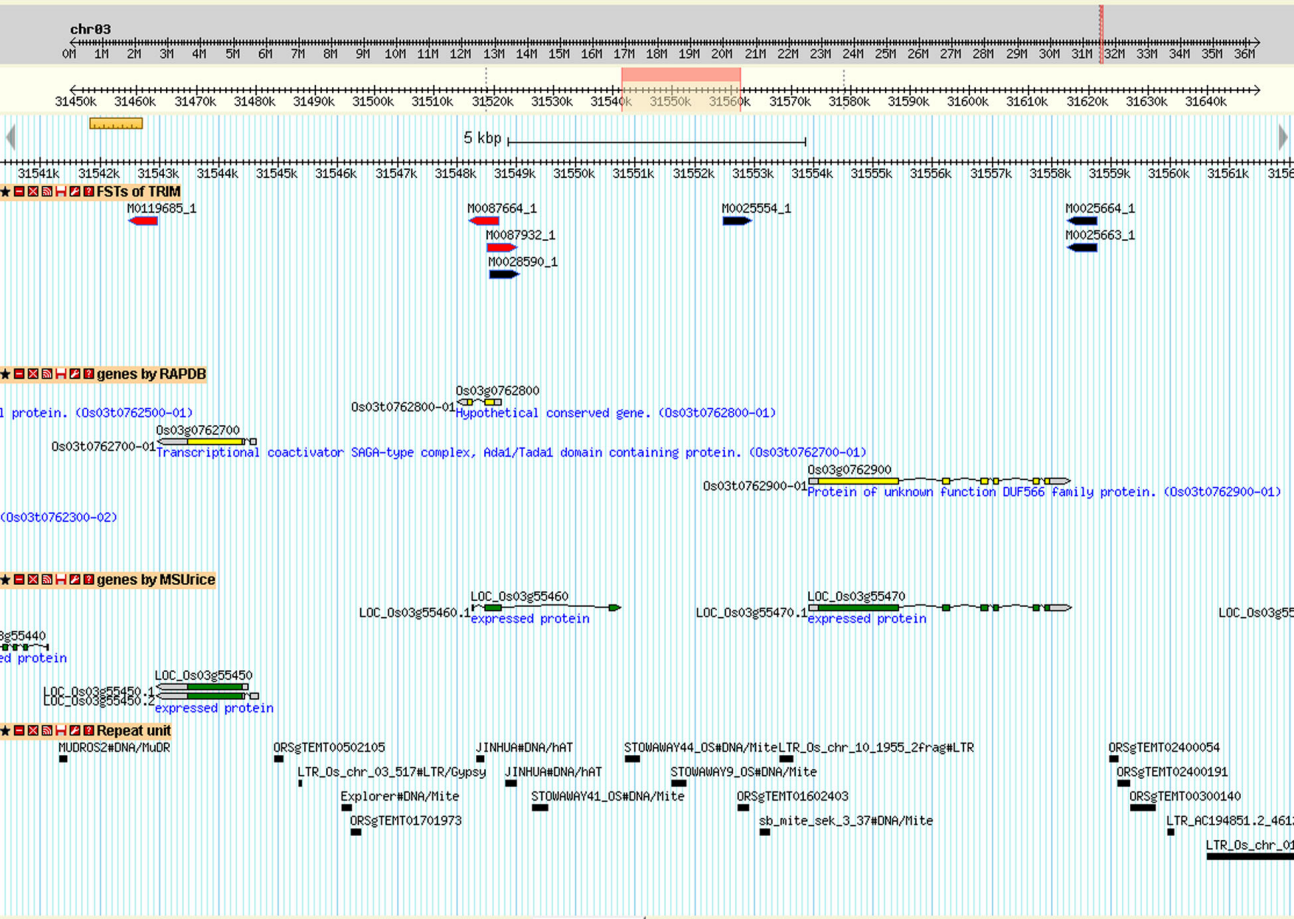
Example of TRIM flanking sequence integration sites revealed by use of genome browsers. The 20-kb region locates at 13_ _781_ _735 to 13_ _801_ _735 bp of chromosome 3. The upper section illustrates the flanking sequence tag in TRIM, the second section genes annotated by RAP-db [8], the third section genes annotated by the MSU Rice database [35], and the last section repeat sequences annotated by MSU Rice.

As of January 2017, we have FST data for 59_ _590 lines. About 47_ _883 of the FSTs showed hits in the rice genome, which are all available in the databases NCBI GSS (library accession: LIBGSS_009952; library name: AS_TRIM_TDNA_B1), RAP-db, RiceGE, OryGenesDB, or TRIM. These integrated events may affect 33_ _402 non-transposable element (non-TE) genes, including 11_ _695 putative knock-out genes and 33_ _298 putative activated genes. The list of the putative knock-out genes is listed in Table S4. Thus, 85.5% of the rice non-TE genes may be affected. In other studies of rice or Arabidopsis insertional mutant populations, about 20% to 30% of the transgenic plants contained a multiple tandem T-DNA array or truncated T-DNA region, so the products of thermal asymmetric interlaced polymerase chain reaction [20] or similar methods [21] did not contain the genome sequence [21]. In TRIM, about one-third of the FSTs feature the same problem. All FST integration sites and possible affected gene regions can be searched on the TRIM website. Fig. 3 shows a 20-kb region at 13.8 Mbp of chromosome 3. The red bar indicates that the integration has triple functions (knock-out, activation, and promoter trapping), and the black bar indicates double functions (knock-out and promoter trapping). Because genes within the 15-kb region upstream or downstream of the integration site might be activated by enhancers in the T-DNA [2], 2 of the 3 genes in the 20-kb region may be knocked out, and all 3 genes may be activated in the 6 TRIM lines integrated in this region.

## Data Validation and Quality Control

### Detailed analysis of TRIM leads to gene discoveries

The rice dwarf mutant *d1*, defective in the α subunit of the heterotrimeric G protein, was proposed to affect gibberellin signal transduction [22]. This *d1* mutant has round seeds and short panicles and is dwarf or semi-dwarf. We used these traits to search the TRIM database and found about 30 lines. We sequenced randomly picked 6 lines and found that M0000625, M0005254, M0001475, and M0033961 had single nucleotide polymorphisms (SNPs) in the *d1* gene region that caused an early translational stop [23]. However, mutations in other unidentified genes should be responsible for the other 2 mutant lines. The previous whole-genome sequencing analysis indicated that rice regenerants and transformants consisted of about 200 SNPs/indel per plant. This number increased to 3- to 10-fold higher in TRIM accessions as there were longer culture periods [23].

Phytohormone strigolactone has been reported with anti-stress functions and is an important topic for research [e.g., reviewed in 24]. Several strigolactone biosynthesis-related genes, such as *d3, d10, d14, d17*, and *d27*, in rice have been cloned, and the loss-of-function mutant showed dwarf tillering phenotypes [24]. There are 90 dwarf tillering TRIM lines, and we performed whole-genome sequencing analysis of 5 randomly picked lines. M0028590, M0079651, and M0084311 had an SNP at *D17* or *D27* that led to an early translational stop. However, M0048349 had a 26.2-kbp deletion containing *D17*, and M0053677 had a 13.8-kbp deletion containing *D14* [23, 25]. Thus, a detailed analysis of these TRIM lines may provide further clues about the regulation of biosynthesis and functions of strigolactone in plants.

Because more than 80% of the rice non-TE genes may be activated in the TRIM population, the specific phenotype is a dominant trait if the gene is activated, and thus it may be screened from the T_1_ or T_2_ generations. With this convenient feature, we joined the international C4-like rice consortium and screened for the vein-spacing mutants. It has been demonstrated that reduced vein spacing, i.e., 2 or 3 mesophyll cells between 2 adjacent bundle sheaths, is one of the specific features for C4 cereal leaves [26, 27]. This screening effort is still in progress, and we expect to eventually identify genes related to reducing vein spacing. Thus, large-scale phenotype screening of the TRIM population followed by segregation analysis with the tagged genes can lead to discovery of novel genes.

### Comparison with other mutant resources

All available rice insertional mutant resources have data on phenomics study of plants grown under field conditions [13, 14, 16]. In addition, they all have about 60 traits screened and recorded. Nipponbare, the *japonica* rice variety used by the International Consortium for Genome Sequencing, was used for the databases OTL [13] and *Tos17* [10], but the Taiwan local *japonica* variety TNG67 was used for TRIM. The 3 endogenous *Tos17* copies of TNG67 stay inactive during the cultured condition [1], and this variety is well adapted to subtropical regions and not sensitive to day length or temperature as compared with Nipponbare. Table 2 shows variations in trait frequency among 3 resources—OTL, *Tos17*, and TRIM. For instance, the *Tos17* population has a very high ratio of dwarf and semi-dwarf traits (18%), which is relatively low in TRIM or OTL resources. The trait frequency of heading, including early, late, or no heading, is very low in TRIM but higher in the other 2 resources. These differences might be related to the function of *Heading date 1* (*Hd1*) and *Early heading date 1* (*Ehd1*) in Nipponbare, which are lost in TNG67 [28]. In addition, TRIM features a higher frequency of dark-green, narrow, short leaf, short panicle, and small grain as compared with the other resources, which shows the relationship among varieties, cultivation locations, and cropping seasons.

**Table 2:** Frequency of trait variation in 3 rice mutant resources

Phenotype sub-category	Lines	TRIM	OTL^a^	*Tos17* ^b^
Yellow leaf	79	0.10	2.35	1.62
Dark-green leaf	3036	3.85	0.10	2.13
Pale-green leaf	1884	2.39	0.07	3.46
Narrow leaf	4880	6.20	0.17	2.76
Short leaf	2350	2.98	0.11	0.08
Dwarf and semi-dwarf	9300	11.81	3.94	18.78
High tiller numbers	1679	2.13	0.28	0.22
Early heading	292	0.37	0.00	3.59
Late heading	251	0.32	1.44	2.49
No heading	180	0.23	0.00	0.19
Short panicle	2188	2.78	0.01	1.50
Small grain	2261	2.87	0.26	0.85
Total number of lines		78_ _769	27_ _832	50_ _000

^a^OTL data from Lorieux et al. 2012 [13].

^b^Data from the *Tos17* website [34]. Also the same as the data from Lorieux et al. 2012 [13].

### Comparison with the 3K rice genomes project

From the joint efforts of International Rice Research Institute (IRRI) and Beijing Genomics Institute, the 3K rice genomes project is a dataset of publically available genome sequences from 3000 rice accessions [29]. In parallel to the sequencing work, IRRI also performed the phenomics analysis of about two-thirds of these lines. The phenotype data of 74 traits, including 59 category types and 15 quantitative traits, are available on the IRRI website [30]. Thus, the 3K rice genomes project provides a vast amount of natural sequence variations in the 3K accessions, as well as plenty of phenotype information. In comparison, an insertional mutant population such as TRIM provides precise FST information; thus the mutated genes are known, i.e., usually one gene for each knock-out line or 3 to 5 genes for each activation line. There are 71 traits, including 3 quantitative ones, in the TRIM population, and about 20% of them are similar to those in the 3K database. Since the number of candidate genes for TRIM mutants is usually small, i.e, one to a few, it provides an efficient tool for the study of functions of specific genes. In comparison, one has to first follow a genome-wide approach, such as using the 3K database, to narrow down the chromosome region where the candidate gene resides to within several Mb, equivalent to a few hundred genes.

## Re-use Potential Beyond Rice Functional Genomics

Although TRIM is a valuable resource for rice functional genomics studies in terms of identification of novel genes (forward genetics) and investigations of function of known genes (reverse genetics), its use is beyond rice functional genomics. First, because of the high synteny between rice and other cereals [31], information from TRIM can be extended to the study of other cereal genes located in chromosome regions sharing synteny with rice. Second, genes studied with TRIM can be used in marker-assisted breeding, which has become the standard in modern agronomical practices. Third, since most of the TRIM mutant phenotypes are generated by activation tagging, the genes identified following the forward genetics approach can be readily used in crop improvement via genetic engineering in which beneficial genes are ectopically expressed, usually driven by a strong constitutive promoter. Finally, recently developed genome editing technology [32] can be used to modify genes whose functions are elucidated with the help of TRIM.

## Data availability

The FST data may be searched by using the genome browser at the RiceGE, RAP-db, OryGenesDB, and TRIM websites. All sequences may also be downloaded from the GSS database at NCBI. The phenomics data are available in Table S3 and at the TRIM website. Table S3 has been deposited in the *GigaScience* database, GigaDB [33].

## Additional files

Additional file 1: Table S1. T_1_ rice lines screened since 2002. Table S2. Classification of observed phenotypes in the rice paddy field. Table S3. Phenomics data for all TRIM lines (Excel file). Table S4. The list of putative knocked-out genes in the TRIM lines (Excel file). Table S5. Supplementary materials authors.

## Abbreviations

FST: flanking sequence tag; GSS = National Center for Biotechnology Information Genome Survey Sequences database; IRRI: International Rice Research Institute; OTL: *Oryza* Tag Line; SNP = single nucleotide polymorphism; TNG67: Tainung 67; TRIM: Taiwan Rice Insertion Mutants.

## Competing interests

The authors declare that they have no competing interests.

## Funding

This project was supported by grants from the National Science and Technology Program (NSTP/AB 96S-1501), Academia Sinica Genomics and Proteomics Integrated Program (098S0030032-AH), and Academia Sinica Investigator Award (100-ASIA) to Y.I.C.H. It was also supported by grants from National Science Council (NSC 103-2321-B-001-049) and Ministry of Science and Technology (MOST 104-2321-B-001-044) to S.M.Y. and (106-2321-B-001-016) to T.H.D.H.

## Author contributions

S.M.Y., T.H.D.H., Y.I.C.H., designed the project; F.J.W., H.P.W., and C.C.W. performed the data analysis; M.H.L., S.F.L., L.J.C., M.J.F., S.C., and I.C.W. led the phenotyping team; Y.I.C.H. wrote the manuscript. All authors have read and approved the final manuscript.

## Supplementary Material

GIGA-D-17-00045_Original-Submission.pdf

GIGA-D-17-00045_Revision-1.pdf

GIGA-D-17-00045_Revision-2.pdf

Response-to-Reviewer-Comments_Original-Submission.pdf

Response-to-Reviewer-Comments_Revision-1.pdf

Reviewer-1-Report-(Original-Submission).pdf

Reviewer-1-Report-(Revision-1).pdf

Reviewer-2-Report-(Original-Submission).pdf

Reviewer-2-Report-(Revision-1).pdf

Reviewer-3-Report-(Original-Submission).pdf

Supplement Tables
