# Supplementary material for: Large-scale phenomics analysis of a T-DNA tagged mutant population
Source: Gigascience. 2017 Jul 13;6(8):1–7. doi: 10.1093/gigascience/gix055 (PMC5570018; doi:10.1093/gigascience/gix055)
Supplement: GIGA-D-17-00045_Revision-1.pdf [file gix055_GIGA-D-17-00045_Revision-1.pdf]

Manuscript Click here to download Manuscript manuscript 0512.docx

Large-scale phenomics analysis of a T-DNA tagged mutant population

Hshin-Ping Wu[1]
hpwu@gate.sinica.edu.tw
Fu-Jin Wei[1]
masker@gate.sinica.edu.tw
Cheng-Chieh Wu[1,2]
ccwu1212@gate.sinica.edu.tw
Shuen-Fang Lo[3,4]
jjpipi@ms11.hinet.net
Liang-Jwu Chen[5]
ljchen@dragon.nchu.edu.tw
Ming-Jen Fan[6]
mjfan@asia.edu.tw
Shu Chen[7]
CShu@tari.gov.tw
Ien-Chie Wen[7]
ICWen@tari.gov.tw
Su-May Yu[3,4,8]
sumay@imb.sinica.edu.tw
Tuan-Hua David Ho[1,4, 8]
tho@gate.sinica.edu.tw
Ming-Hsin Lai[9]
mhlai@tari.gov.tw
Yue-ie C Hsing[1, 10*]
[*] Corresponding author
bohsing@gate.sinica.edu.tw

[1]Institute of Plant and Microbial Biology, Academia Sinica, Nankang, Taipei 115, Taiwan
[2]Institute of Plant Biology, National Taiwan University, Taipei 106, Taiwan
[3]Institute of Molecular Biology, Academia Sinica, Taipei 115, Taiwan
[4]Agricultural Biotechnology Center, National Chung Hsing University, Taichung 402, Taiwan

[5]Institute of Molecular Biology, National Chung Hsing University, Taichung 402, Taiwan

[6]Department of Biotechnology, Asia University, Taichung 413, Taiwan

[7]Plant Germplasm Division, Taiwan Agricultural Research Institute, Taichung 413, Taiwan

[8]Department of Life Sciences, National Chung Hsing University, Taichung 402, Taiwan

[9]Crop Science Division, Taiwan Agriculture Research Institute, Taichung 413, Taiwan

[10]Department of Agronomy, National Taiwan University, Taipei 106, Taiwan

Abstract

Background: Rice, *Oryza sativa* L., is one of the most important crops in the world. With the rising world population, feeding people in a more sustainable and environment-friendly way becomes increasingly important. Therefore, rice research community needs to share resources to better understand functions of rice genes that are the foundation for future agricultural biotechnology development, and one way to achieve this goal is via the extensive study of insertional mutants.

Findings: We have constructed a large rice insertional mutant population in a *japonica* rice variety, Tainung 67. The collection contains about 93,000 mutant lines, among them 85% with phenomics data and 65% with flanking sequence data. We screened the phenotypes of 12 individual plants for each line grown under field conditions according to 68 subcategories and 3 quantitative traits. Both phenotypes and integration sites are searchable in the database at Taiwan Rice Insertional Mutants Database (http://trim.sinica.edu.tw).

Conclusions: Detailed analyses of phenomics data, T-DNA flanking sequences and whole-genome sequencing data for rice insertional mutants can lead to the discovery of novel genes. In addition, studies of mutant phenotypes can reveal relationships among varieties, cultivation locations, and cropping seasons.

Keywords:

Flanking sequence, large-scale phenomics, rice, sequence analysis, T-DNA insertional mutants

Mutation resource description

Purpose of data acquisition

With the rising world population, feeding people in a more sustainable and environment-friendly way becomes increasingly important. Toward this end, the rice research community needs to share resources to better understand functions of rice genes and their roles in phenotypes, especially genes encoding important agronomic traits.

An important way to define the function of a novel gene is to abolish or activate its expression by using a tagged sequence such as T-DNA [e.g., 1, 2], *Tos17* [3], *Ac/Ds* [e.g., 4] or *Spm* [e.g., 5]. Many research groups have established rice insertional mutant resources and provided flanking sequence tag (FST) information for these mutant lines. As of October 2016, about 450,000 integration sites were available in public databases, such as RiceGE (http://signal.salk.edu/cgi-bin/RiceGE), OryGenesDB (http://orygenesdb.cirad.fr), RAPdb (http://rapdb.dna.affrc.go.jp/), and NCBI Genome Survey Sequences (GSS, https://www.ncbi.nlm.nih.gov/). Several recent papers [6, 7, 8] reviewed these rice mutant resources and their applications.

However, much less effort has been devoted to phenomics analyses for these mutant lines. For instances, there are 27,832 phenotype records for the *Oryza* Tag Line (OTL) resource in France [9], 50,000 for the *Tos17* resource in Japan [10], 31,000 for the Rice Mutant Database in China [11], and 78,769 for the Taiwan Rice Insertional Mutants (TRIM) database in Taiwan [2, 12]. Thus, the total numbers with phenomics information is less than half of the FST data.

Using T-DNA as a tagged sequence with a local *japonica* rice variety, Tainung 67 (TNG67), we prepared a large rice insertional mutant resource in Taiwan — TRIM [1, 2, 12]. The T-DNA sequence we used provided three functions: gene knock-out, gene activation, and promoter trapping. We started the work in 2002 and have accumulated many mutant lines as well as phenomics and flanking sequence data. All of these data are searchable at the TRIM website (http://trim.sinica.edu.tw). With an application to the T-DNA Tagged Rice Service Center (http://tdna.bts.asia.edu.tw/index2.html), researchers can receive 30 T_2 seeds for each line requested.

Many TRIM lines have been used for several forward and reverse genetics analyses [reviewed in 2]. For instance, we studied the relationship between flanking sequences and phenomics data by offspring segregation, gene expression, and

overexpression, and confirmed three genes controlling the large-grain trait and seed yield [2]. Thus, detailed data-mining of both flanking sequence and phenomics data may provide useful information for investigating important agronomic traits. Here we report the current status of this valuable genetic resource and discuss the efficient way to use it as well as differences among three rice phenomics populations.

Phenomics data description and analysis

Breeders perform phenotyping in an experimental field for genetically modified (GM) plants

Since the *japonica* rice variety TNG67 is not sensitive to day length and temperature, it can be grown in two cropping seasons each year. The total rice growing time in Taiwan each year is about 10 months, with two cropping seasons of about 4 to 5 months each. The TRIM line numbers are assigned according to the chronologic order of their generation. The smallest number is M00000031 and the highest is M00127550. Our seed collection contains 86,310 lines with T_1 seeds (collected from the T_0 plants), and 78,757 lines with T_2 seeds. While we propagated the T_2 seeds, we performed phenomics screening of T_1 plants grown in the field (Nungliang # 0961050799, issued at August 3rd, 2007), which has been carried out since the second cropping season in 2002 (Table S1). These T_1 plants were grown in the field in a single-seed-descent manner.

In total, 68 traits, belonging to 11 categories (Table S2), were screened by five well-trained breeders. In addition, three quantitative traits were recorded for each line, including plant height, panicle number and heading date. Six seed traits screened for about 10,000 lines, including germination rate, seed length, seed width, seed height, 100-seed weight, and seed length/width ratio, have also been recorded. Detailed methods for phenomics studies were published in a protocol-type review [13].

Altogether, 92,644 lines have been grown; 62,375 did not have any detectable mutant traits during growth under normal field conditions. For lines with clearly visible mutant phenotypes, 14,129, 1,993, 244, 26, and 2 lines contained one, two, three, four, and five groups of mutant traits, respectively; that is, 17.7% of the T_1 population contained mutated traits. In our previous paper on phenomics [12], the mutation percentage was estimated at 17.9% in a 22,665 T_1 population. Thus, the mutation frequency remained similar over the decade. The 68 traits of all TRIM lines

can be searched online at the TRIM website (Figure 1). The phenomics data for all TRIM lines are available in Table S3.

Among the lines screened, the most frequently occurring phenotype categories are plant stature and leaf morphology (Table 1). Together, about half of the mutant traits belong to these two categories. The least-occurring categories are heading date, development and lesion mimic: less than 5% of the mutant traits belong to these three categories. Figure 2 shows the four most frequent phenotype categories. The plant stature category contains 9 traits (Figure 2A), and among them dwarf, thin culm and lazy canopy categories constitute more than 90% of mutants in this category. Leaf morphology has 13 traits (Figure 2B) with the most frequent being narrow, short, rolled, long and wide leaf. Leaf color has 10 traits (Figure 2C) with the most frequent being dark-green, pale-green, bluish-green and striped leaf. Notably, dark-green leaf represents about half of the traits. Panicle mutation has 12 traits (Figure 2D) with the most frequent being short, sparse, and dense panicle and neck leaf. Table S3 lists the phenomics records for all the TRIM lines we have observed to date. These are also searchable at the TRIM website using the phenotype trait, as shown in Figure 1.

Flanking sequence data description

As of January 2017, we have FST data for 59,590 lines. About 47,833 of the FSTs showed hits in the rice genome, which are all available in the databases NCBI GSS (library accession: LIBGSS_009952; library name: AS_TRIM_TDNA_B1), RAP-db, RiceGE, or TRIM. These integrated events may affect 33,402 non-transposable element (non-TE) genes, including 11,695 putative knock-out genes and 33,298 putative activated genes. The list of the putative knocked-out genes is listed in Table S4. Thus, 85.5% of the rice non-TE genes may be affected. In other studies of rice or Arabidopsis insertional mutant populations, about 20% to 30% of the transgenic plants contained a multiple tandem T-DNA array or truncated T-DNA region, so the products of TAIL-PCR [14] or similar methods [15] did not contain the genome sequence [15]. In TRIM, about one-third of the FSTs feature the same problem. All FST integration sites and possible affected gene regions can be searched in the TRIM website. Figure 3 shows a 20-kb region at 13.8 Mbp of chromosome 3. The red bar indicates that the integration has triple functions (knockout, activation and promoter trapping) and the black bar indicates double functions (knockout and promoter

trapping). Because genes within the 15-kb region upstream or downstream of the integration site might be activated by enhancers in the T-DNA [2], two of the three genes in the 20-kb region may be knocked out and all three genes may be activated in the six TRIM lines integrated in this region.

Findings

Detailed analysis of TRIM leads to gene discoveries

The rice dwarf mutant *d1*, defective in the α subunit of the heterotrimeric G protein, was proposed to affect gibberellin signal transduction [16]. This *d1* mutant has round seeds and short panicles and is dwarf or semi-dwarf. We used these traits to search the TRIM database and found about 30 lines. We sequenced randomly-picked six lines and found that M0000625, M0005254, M0001475, and M0033961 had single nucleotide polymorphisms (SNPs) in the *d1* gene region that caused an early translational stop [17]. However, mutations in other unidentified genes should be responsible for the other two mutant lines. The previous whole-genome sequencing analysis indicated that rice regenerants and transformants consisted of about 200 SNP/indel per plant. This number increased to 3- to 10-fold higher in TRIM accessions as there were longer cultured period [17].

Phytohormone strigolactone has been reported with anti-stress functions, and is an important topic for research [e.g., reviewed in 18]. Several strigolactone biosynthesis-related genes, such as *d3, d10, d14, d17,* and *d27,* in rice have been cloned, and the loss-of-function mutant showed dwarf tillering phenotypes [18]. There are 90 dwarf tillering TRIM lines, and we performed whole genome sequencing analysis of 5 randomly-picked lines. M0028590, M0079651 and M0084311 had an SNP at *D17* or *D27* that led to an early translational stop. However, M0048349 had a 26.2-kbp deletion containing *D17* and M0053677 had a 13.8-kbp deletion containing *D14* [17, 19]. Thus, the detailed analysis of these TRIM lines may provide further clues about the regulation of biosynthesis and functions of strigolactone in plants.

Comparison with other mutant resources

All available rice insertional mutant resources have data on phenomics study of plants grown under field conditions [9, 10, 12]. In addition, they all have about 60 traits screened and recorded. Nipponbare, the *japonica* rice variety used by the international consortium for genome sequencing, was used for the databases OTL [9] and *Tos17*

[10], but the Taiwan local *japonica* variety TNG67 was used for TRIM. The three endogenous *Tos17* copies of TNG67 stay inactive during the cultured condition [1], and this variety is well adapted to subtropical regions and not sensitive to day length or temperature as compared with Nipponbare. Table 2 shows variations in trait frequency among three resources — OTL, *Tos17* and TRIM. For instance, the *Tos17* population has a very high ratio of dwarf and semi-dwarf traits (18%), which is relatively low in TRIM or OTL resources. The trait frequency of heading, including early, late or no heading, is very low in TRIM but higher in the other two resources. These differences might be related to the function of *Heading date 1* (*Hd1*) and Early *Heading date 1* (*Ehd1*) in Nipponbare, which are lost in TNG67 [20]. In addition, TRIM features a higher frequency of dark-green, narrow, and short leaf, short panicle, and small grain as compared with the other resources, which shows relationship among varieties, cultivation locations, and cropping seasons.

Availability and requirements

Data availability

The FST data may be searched by using the genome browser at RiceGE, RAP-db, and TRIM websites. All sequences may also be downloaded from the GSS database in NCBI. The phenomics data are available in Table S3 and at the TRIM website. Table S3 has been deposited in the GigaScience database (GigaDB).

Additional files

Additional file 1: Table S1. T_1 rice lines screened since 2002

Table S2. Classification of observed phenotypes in the rice paddy field

Table S3. Phenomics data for all TRIM lines (Excel file)

Table S4. The list of putative knocked-out genes in the TRIM lines (Excel file)

Table S5. Supplementary materials authors

List of abbreviations

FST: flanking sequence tags; OTL: *Oryza* Tag Line; TNG67: Tainung 67; TRIM: Taiwan Rice Insertion Mutants.

Ethics approval and consent to participate

No applicable.

Consent for publication

The authors declare no competing interests.

Competing interests

The authors declare no competing interests.

Authors' contributions

SMY, THDH, YICH designed the project; FJW, HPW, CCW performed the data analysis; MHL, SFL, LJC, MJF, SC, ICW led the phenotyping team; YICH wrote the manuscript. All authors have read and approved the final manuscript.

Acknowledgements

The project was supported by grants from Academia Sinica, Council of Agriculture, Taiwan Agricultural Research Institute, and the Ministry of Science and Technology, Taiwan, to SMY, THDH, MHL and YICH. The team expresses thanks to all members listed in Table S5 (Supplementary materials authors). We thank Laura Smales (BioMedEditing, Toronto, Canada) for English editing.

Received: Revised: Accepted:

References

1. Hsing Y-I, Chern C-G, Fan M-J, Lu P-C, Chen K-T, Lo S-F, et al. A rice gene activation/knockout mutant resource for high throughput functional genomics. Plant molecular biology. 2007;63 3:351-64.
2. Lo S-F, Fan M-J, Hsing Y-I, Che L-J, Chen S, Wen I-C, et al. Genetic resources offer efficient tools for rice functional genomics research. Plant, Cell & Environment. 2016;39 5:998-1013.
3. Miyao A, Tanaka K, Murata K, Sawaki H, Takeda S, Abe K, et al. Target site specificity of the *Tos17* retrotransposon shows a preference for insertion within genes and against insertion in retrotransposon-rich regions of the genome. The Plant cell. 2003;15 8:1771-80.
4. He C, Dey M, Lin Z, Duan F, Li F and Wu R. An efficient method for producing an indexed, insertional-mutant library in rice. Genomics. 2007;89 4:532-40.
5. Kumar CS, Wing RA and Sundaresan V. Efficient insertional mutagenesis in rice using the maize En/Spm elements. The Plant Journal. 2005;44 5:879-92.
6. Droc G, An G, Wu C, Hsing Y-iC, Hirochika H, Pereira A, et al. Mutant Resources for Functional Analysis of the Rice Genome. In: Zhang Q and Wing RA, editors. Genetics and Genomics of Rice. New York: Springer; 2013. p. 81-115.
7. Wei F-J, Droc G, Guiderdoni E and Yue-ie CH. International consortium of rice mutagenesis: resources and beyond. Rice. 2013;6 1:39.
8. Wang N, Long T, Yao W, Xiong L, Zhang Q and Wu C. Mutant resources for the functional analysis of the rice genome. Molecular Plant. 2013;6 3: 596-604.
9. Lorieux M, Blein M, Lozano J, Bouniol M, Droc G, Diévart A, et al. In-depth molecular and phenotypic characterization in a rice insertion line library facilitates gene identification through reverse and forward genetics approaches. Plant biotechnology journal. 2012;10 5:555-68.
10. Miyao A, Iwasaki Y, Kitano H, Itoh J-I, Maekawa M, Murata K, et al. A large-scale collection of phenotypic data describing an insertional mutant population to facilitate functional analysis of rice genes. Plant molecular biology. 2007;63 5:625-35.
11. Zhang J, Li C, Wu C, Xiong L, Chen G, Zhang Q, et al. RMD: a rice mutant database for functional analysis of the rice genome. Nucleic acids research. 2006;34 suppl 1:D745-D8.
12. Chern C-G, Fan M-J, Yu S-M, Hour A-L, Lu P-C, Lin Y-C, et al. A rice

phenomics study—phenotype scoring and seed propagation of a T-DNA insertion-induced rice mutant population. Plant molecular biology. 2007;65 4:427-38.

13. Chern C-G, Fan M-J, Huang S-C, Yu S-M, Wei F-J, Wu C-C, et al. Methods for rice phenomics studies. Plant Reverse Genetics: Methods and Protocols. 2011:129-38.

14. Liu Y-G and Whittier RF. Thermal asymmetric interlaced PCR: automatable amplification and sequencing of insert end fragments from P1 and YAC clones for chromosome walking. Genomics. 1995;25 3:674-81.

15. Li R, Quan S, Yan X, Biswas S, Zhang D and Shi J. Molecular characterization of genetically-modified crops: Challenges and strategies. Biotechnology Advances. 2017;35 2:302-9.

16. Ashikari M, Wu J, Yano M, Sasaki T and Yoshimura A. Rice gibberellin-insensitive dwarf mutant gene Dwarf 1 encodes the α-subunit of GTP-binding protein. Proceedings of the National Academy of Sciences. 1999;96 18:10284-9.

17. Wei FJ, Kuang LY, Oung HM, Cheng SY, Wu HP, Huang LT, et al. Somaclonal variation does not preclude the use of rice transformants for genetic screening. The Plant journal. 2016;85 5:648-59.

18. Al-Babili S and Bouwmeester HJ. Strigolactones, a novel carotenoid-derived plant hormone. Annual review of plant biology. 2015;66:161-86.

19. Wei F-J, Tsai Y-C, Hsu Y-M, Chen Y-A, Huang C-T, Wu H-P, et al. Lack of genotype and phenotype correlation in a rice T-DNA tagged line is likely caused by introgression in the seed source. PloS one. 2016;11 5:e0155768.

20. Wei F-J, Tsai Y-C, Wu H-P, Huang L-T, Chen Y-C, Chen Y-F, et al. Both *Hd1* and *Ehd1* are important for artificial selection of flowering time in cultivated rice. Plant Science. 2016;242:187-94.

Figure legends

Figure 1 Example of search results for the trait "yellow leaf" in the TRIM resource

Figure 2 Trait percentages for the four high abundant phenotype categories in the TRIM resource. A) plant stature; B) leaf morphology; C) leaf color; and D) panicle.

Figure 3 Example of TRIM flanking sequence integration sites revealed by use of genome browsers. The 20-kb region locates at 13,781,735 to 13,801,735 bp of chromosome 3. The upper section illustrates the flanking sequence tag in TRIM, the second section genes annotated by RAP-db (http://rapdb.dna.affrc.go.jp/), the third section genes annotated by the MSU Rice database (http://rice.plantbiology.msu.edu/index.shtml), and the last section repeat sequences annotated by MSU Rice.

 Click here to download Table Table 1.docx

Table 1 Frequency of phenotypes in the Taiwan Rice Insertional Mutant (TRIM) library

**Categories**	**Lines**	**Percentage**
Plant stature	11,059	24.51
Leaf Morphology	9,107	20.18
Leaf Color	5,764	12.77
Fertility	5,170	11.46
Panicle	5,144	11.40
Grain	2,862	6.34
Glume	2,433	5.39
Tiller Position	1,635	3.62
Heading Date	719	1.59
Lesion mimic	643	1.42
Development	590	1.31
**Total number of lines**	45,126	45,126
**Percentage**		100.00

Table 2 Frequency of trait variation in three rice mutant resources

Phenotype sub-category	Lines	TRIM	OTL*	*Tos17'*
**Yellow leaf**	79	0.10	2.35	1.62
**Dark-green leaf**	3,036	3.85	0.10	2.13
**Pale-green leaf**	1,884	2.39	0.07	3.46
**Narrow leaf**	4,880	6.20	0.17	2.76
**Short leaf**	2,350	2.98	0.11	0.08
**Dwarf and semi-dwarf**	9,300	11.81	3.94	18.78
**High tiller numbers**	1,679	2.13	0.28	0.22
**Early heading**	292	0.37	0.00	3.59
**Late heading**	251	0.32	1.44	2.49
**No heading**	180	0.23	0.00	0.19
**Short panicle**	2,188	2.78	0.01	1.50
**Small grain**	2,261	2.87	0.26	0.85
**Total number of lines**		78,769	27,832	50,000

*: Oryza Tag Lines (OTL) data from Lorieux et al., 2012.

': data from the Tos17 website (https://tos.nias.affrc.go.jp/phenotype/). Also the same as the data from Lorieux et al., 2012.

Figure 1

Click here to download Figure fig 1.PNG

The list of TRIM lines with the trait :

Yellow leaf	M0004082	M0004714	M0004884	M0004918	M0006187	M0006335	M0008462	M0009023	M0009285	M0009618
	M0009734	M0013883	M0019315	M0021075	M0021115	M0021231	M0026438	M0032937	M0036382	M0059526
	M0060863	M0063025	M0063955	M0064728	M0064729	M0065518	M0066601	M0067278	M0067667	M0067937
	M0074871	M0076123	M0076204	M0076493	M0077220	M0077389	M0078493	M0078865	M0081834	M0083280
	M0083918	M0084253	M0087161	M0089231	M0091819	M0096284	M0096293	M0096502	M0097127	M0097296
	M0097380	M0099038	M0099657	M0099790	M0101549	M0104894	M0105660	M0105979	M0106234	M0106698
	M0108929	M0110321	M0112906	M0114250	M0114516	M0114781	M0114886	M0115772	M0116153	M0116600
	M0117353	M0118630	M0119256	M0119527	M0121347	M0124856	M0124895	M0127317	M0127526	

Figure 2

Click here to download Figure fig 2.tif

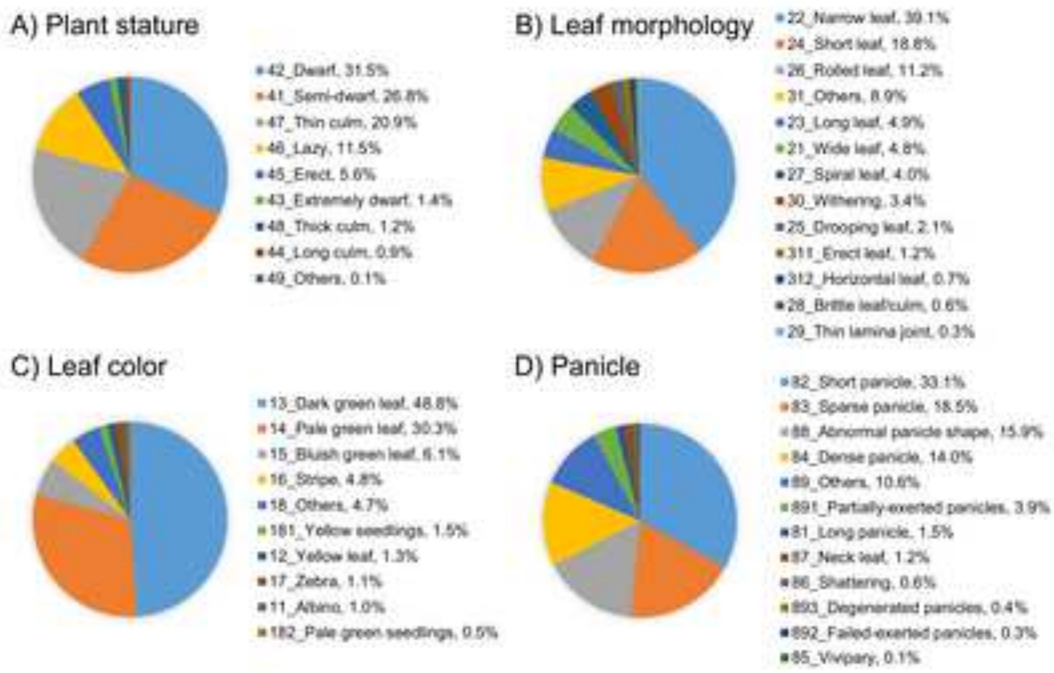

Figure 3

Click here to download Figure fig 3.tif

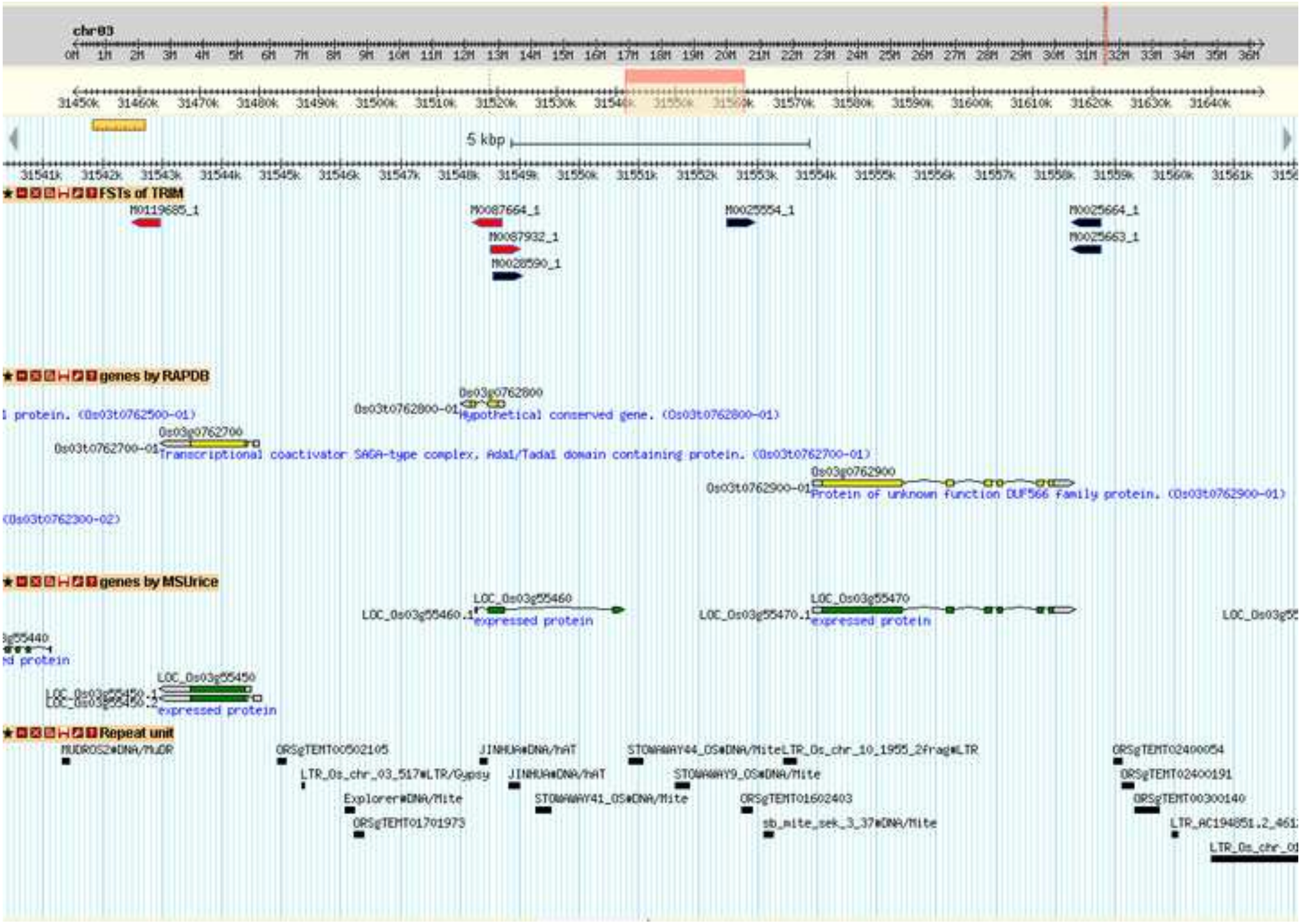

Click here to access/download
Supplementary Material
Table S1 The T1 lines screened since 2002.docx

Click here to access/download
Supplementary Material
Table S2 Classification of observed phenotypes.docx

Supplementary Material Table S3

Click here to access/download
Supplementary Material
TRIM_Table_S3.xlsx

Supplementary Table 4

Click here to access/download
Supplementary Material
Table_S4_konckout.xlsx

Supplementary Table 5

Click here to access/download
Supplementary Material
Table S5 Supplementary materials authors.docx
