# Supplementary material for: Large-scale phenomics analysis of a T-DNA tagged mutant population
Source: Gigascience. 2017 Jul 13;6(8):1–7. doi: 10.1093/gigascience/gix055 (PMC5570018; doi:10.1093/gigascience/gix055)
Supplement: Supplement Tables [file gix055_Supplement-Tables.zip › Table S1 The T1 lines screened since 2002.docx]

**Table S1 The T_1_ lines screened since 2002**

| **Cropping season** | **T_1_ lines screened*** | **Cropping season** | **T_1_ lines screened** |
| --- | --- | --- | --- |
| 2002-2 | 500 | 2009-2 | 5000 |
| 2003-1 | 2498 | 2010-1 | 2700 |
| 2003-2 | 2700 | 2010-2 | 2799 |
| 2004-1 | 5000 | 2011-1 | 2398 |
| 2004-2 | 4975 | 2011-2 | 1500 |
| 2005-1 | 5000 | 2012-1 | 1201 |
| 2005-2 | 5000 | 2012-2 | 1000 |
| 2006-1 | 4998 | 2013-1 | 969 |
| 2006-2 | 5020 | 2013-2 | 616 |
| 2007-1 | 4994 | 2014-1 | 671 |
| 2007-2 | 4992 | 2014-2 | 699 |
| 2008-1 | 4962 | 2015-1 | 677 |
| 2008-2 | 4987 | 2015-2 | 678 |
| 2009-1 | 6030 | 2016-1 | 614 |
|  |  | 2016-2 | 614 |

*Twelve single-seed-descent plants were grown in the genetically modified field for phenomics screening since 2002.
