# Supplementary material for: Large-scale phenomics analysis of a T-DNA tagged mutant population
Source: Gigascience. 2017 Jul 13;6(8):1–7. doi: 10.1093/gigascience/gix055 (PMC5570018; doi:10.1093/gigascience/gix055)
Supplement: Supplement Tables [file gix055_Supplement-Tables.zip › Table S2 Classification of observed phenotypes.docx]

**Table S2 Classification of observed phenotypes in the rice paddy field**

| **Sub-category** | |  |  |  |
| --- | --- | --- | --- | --- |
| Development | Germination rate | | Plant Stature | Thick culm |
| Development | Lethal | | Plant Stature | Others |
| Development | Abnormal plants | | Lesion | Lesion mimic |
| Development | Weak | | Tiller position | High tiller position |
| Leaf Color | Albino | | Tiller position | Low tiller position |
| Leaf Color | Yellow leaf | | Tiller position | Monoculm |
| Leaf Color | Dark green leaf | | Tiller position | Others |
| Leaf Color | Pale green leaf | | Heading Date | Early heading |
| Leaf Color | Bluish green leaf | | Heading Date | Late heading |
| Leaf Color | Stripe | | Heading Date | No heading |
| Leaf Color | Zebra | | Glume | Abnormal hull |
| Leaf Color | Others | | Glume | Abnormal floral organ |
| Leaf Color | Yellow seedlings | | Glume | With awn |
| Leaf Color | Pale green seedlings | | Glume | Abnormal hull |
| Leaf Morphology | Wide leaf | | Glume | Abnormal hull color |
| Leaf Morphology | Narrow leaf | | Panicle | Long panicle |
| Leaf Morphology | Long leaf | | Panicle | Short panicle |
| Leaf Morphology | Short leaf | | Panicle | Sparse panicle |
| Leaf Morphology | Drooping leaf | | Panicle | Dense panicle |
| Leaf Morphology | Rolled leaf | | Panicle | Vivipary |
| Leaf Morphology | Spiral leaf | | Panicle | Shattering |
| Leaf Morphology | Brittle leaf/culm | | Panicle | Neck leaf |
| Leaf Morphology | Thin lamina joint | | Panicle | Abnormal panicle shape |
| Leaf Morphology | Withering | | Panicle | Others |
| Leaf Morphology | Others | | Panicle | Partially-exerted panicles |
| Leaf Morphology | Erect leaf | | Panicle | Failed-exerted panicles |
| Leaf Morphology | Horizontal leaf | | Panicle | Degenerated panicles |
| Plant Stature | Semi-dwarf | | Fertility | Sterile |
| Plant Stature | Dwarf | | Fertility | Low fertility |
| Plant Stature | Extremely dwarf | | Grain | Large grain |
| Plant Stature | Long culm | | Grain | Small grain |
| Plant Stature | Erect | | Grain | Slender grain |
| Plant Stature | Lazy | | Grain | Others |
| Plant Stature | Thin culm | | Grain | Round grain |
