# Supplementary material for: Large-scale phenomics analysis of a T-DNA tagged mutant population
Source: Gigascience. 2017 Jul 13;6(8):1–7. doi: 10.1093/gigascience/gix055 (PMC5570018; doi:10.1093/gigascience/gix055)
Supplement: Supplement Tables [file gix055_Supplement-Tables.zip › Table S5 Supplementary materials authors.docx]

**Table S5 Supplementary materials authors**

Hshin-Ping Wu^1^, Ku-Ting Chen^2,^ Shu Chen^3^, Liang-Jwu Chen^4,5^, Chyr-Guan Chern^6^, Ming-Jen Fan^7^, Ai-Ling Hour^8^, Tuan-Hua David Ho^1,4,9^, Sheng-Chung Huang^3^, Mirng-Jier Jiang^2,4^, Woei-Shyuan Jwo^6^, Yih-Cheng Kuo^6^, Ming-Hsing Lai^6^, Hsing-Fang Lee^1^, Charng-Pei Li^6^, Lung-Sheng Li^6^, Su-Mien Li^6^, Yao-Cheng Lin^10^, Shuen-Fang Lo^2,4^, Po-Chang Lu^1^, Arunee Trisiriroj^1^, Ching-Shan Tseng^10^, Ien-Chie Wen^3^, Fu-Jin Wei^1^, Chiu-Kai Wey^3^, Cheng-Chieh Wu^2^, Chen-Chia Wu^2^, Tung-Lung Yang^1^, Hsing-Mu Yen^6^, Lin-Chih Yu^2^, Su-May Yu^2, 4, 9^, Yue-Ie C. Hsing^1,11^

^1^Institute of Plant and Microbial Biology, Academia Sinica, Taipei, Taiwan

^2^Institute of Molecular Biology, Academia Sinica, Taipei, Taiwan

## ^3^Plant Germplasm Division, Taiwan Agricultural Research Institute, Wufeng, Taichung, Taiwan

^4^Agricultural Biotechnology Center, National Chung Hsing University, Taichung, Taiwan

^5^Institute of Molecular Biology, National Chung Hsing University, Taichung, Taiwan, ROC.

^6^Crop Science Division, Taiwan Agricultural Research Institute, Taichung, Taiwan

^7^Department of Biotechnology and Bioinformatics, Asia University, Taichung, Taiwan

^8^Department of Life Science, Fu-Jen University, Taipei, Taiwan

^9^ Department of Life Sciences, National Chung Hsing University, Taichung, Taiwan, ROC

^10^Agricultural Biotechnology Research Center, Taipei, Taiwan

^11^Department of Agronomy, National Taiwan University, Taipei, Taiwan
