# Supplementary material for: Large-scale phenomics analysis of a T-DNA tagged mutant population
Source: Gigascience. 2017 Jul 13;6(8):1–7. doi: 10.1093/gigascience/gix055 (PMC5570018; doi:10.1093/gigascience/gix055)
Supplement: Response-to-Reviewer-Comments_Original-Submission.pdf [file gix055_Response-to-Reviewer-Comments_Original-Submission.pdf]

Author's Response To Reviewer Comments

Reviewer #1: The study by Wu et al. has constructed a large insertional mutant population (93,000 lines) in a photoperiod-neutral japonica rice variety. The authors have genotyped and phenotyped the mutant population, providing phenomics data for 85% of all mutant lines and flanking sequence data for 65% of them. The phenotyping is fairly systematic, including 68 subcategories and 3 quantitative traits. This study has established an important genetic resource, and the phenotypic data are very valuable to rice researchers worldwide.

The manuscript is well written and readers can easily follow the paper structure.

However, the study needs to be significantly improved before it can be accepted. Following are my comments
1. The authors gave examples of different mutants. The phenotypes of some mutants are caused by somatic mutations during the tissue culture process, rather than the T-DNA insertions. The authors should estimate the copy number of Tos17 and the number of somatic mutations in each line and provide information about what percentage of the observed phenotype is possibly caused by the T-DNA insertion.
Thank you very much for the suggestion. The information about Tos17 and somatic mutations were published previously (Ref. 1, 2, 12, 17, 19). Basically, we have shown that Tos17 in the TNG67 variety we used did not increase in copy number after going through the tissue culture-regeneration process, the level of somatic mutations in TNG67 were also estimated using NGS data. We added those information, along with the references, in the text.

2. The authors compared their resource with other mutant collections. The percentage, for example, of small grain, is so different among resources. The authors need provide more explanations.
We used the TNG67 variety, and the other two collections were with the Nipponbare variety. The genetic background of these varieties is quite different. In addition, the rice plants have one growing season a year for regions where Nipponbare grows, but TNG67 plants can grow in two seasons a year for our collection because we are in a subtropical region and TNG67 is insensitive to photoperiod. Thus, we suggest that genotype, environment, and their interactions may explain why the frequency of several traits differs greatly among resources.

3. Table S3. List affected genes in each line if available. This way will significantly facilitate users.
Thank you very much for the suggestion. We add one more Excel file (Table S4). All putative knock-out genes are listed.

4. Provide Supplemental Methods for resequencing and SNP detection as well as accession numbers of resequence data.
All of the information, including the NGS analysis, accession number, SNP and indel detection and their frequencies, were published in one of our previous papers (Ref. 17, 19).

5. TRIM site. I tried to apply for a new account but was denied. Simply remove the user login requirement.

We constructed the old version TRIM website in 2007, with which users have to register before login. Recently, we generated a new version before we submitted this manuscript and registration is no longer required now. We are sorry that we did not indicate carefully in the previous version of this manuscript. Please visit our website again. Users do not have to register for searching the current FST and phenotype database.

6. Double check the reference format, such as the second one.
Thank you very much for the reminder. We improved it in the current version of this manuscript.

Reviewer #2: I have few questions and concerns before publication of this manuscript:

Please mention permit number for growing GM crops in the field.
Yes, it is Nungliang # 0961050799, and was issued August 3, 2007. We added the information in the current version of the manuscript.

L57 write the name of 3 quantitative traits.
This is an abstract and there is restriction on word count. We did mention it in the manuscript.

L58 Write the name of database?
Yes, we added it in the revision.

L101 please mention names of these 3 genes.
They were in the previous publication (Ref. 2). We are characterizing these genes in detail and will submit more manuscripts on these genes.

L115-116 "Our seed collection contains 86,310 lines with T1 seeds (collected from the T0 plants), and 78,757 lines with T2 seeds" Are these T2 seeds part of 86,310 lines with t1 seeds?
Yes, 78,757 lines with T2 seeds are part of 86,310 lines. Overall, 92,644 lines have T1 or T2 seeds. 86,310 lines of these have T1 seeds and 78,757 lines of these have T2 seeds. (L126: Altogether, 92,644 lines grown.)

L116-117 "While we propagated the T2 seeds, we performed phenomics screening of T1 plants grown in the field" Were the propagation of T2 seeds and T1 phenotyping done during same growth season? How T2 seeds were selected? What was the purpose for the propagation of T2 seeds?
Yes, the propagation of T2 seeds and T1 phenotyping were always done during the same growing season. If the T1 plants did not have any mutant phenotype, the T2 seeds were collected together. If the T1 plants showed a mutant phenotype, then the T2 seeds were harvested into several groups according to the variations of phenotypes, for example, M0121616 has three groups of T2 seeds – M0121616-A, M0121616-B, and M0121616-C. All the detailed procedures were mentioned in our previous protocol papers (Ref. 1, 12, 13). We performed propagation of T2 seeds so that there are enough seeds for gene function studies.

L150-153 "About 47,833 of the FSTs showed hits in the rice genome, which are all available in the databases NCBI GSS, RAP-db, RiceGE, or TRIM." are these all FST available in all databases?

All the FSTs are downloadable at the NCBI GSS database (library accession: LIBGSS_009952; library name: AS_TRIM_TDNA_B1). Once we uploaded new FSTs, we always sent an email to RAP-db and Rice GE asking them to update the TRIM information. Of course, we also updated our TRIM database. However, these three databases are basically genome browsers, so users may search and look at nearby genes. We cannot download the FSTs from RAP-db, RiceGE, or TRIM.

L162-163 'The red bar indicates that the integration has triple functions (knockout, activation and promoter trapping)" How can an integration causes knockout as well as activation at the same time?
All knockout or activated genes are based on prediction. Therefore, RT-PCR has to be used to confirm whether they are knocked out or activated. Depending on the T-DNA integrated position, they may be knocked out or activated.

The mutants with the red bar indicate the T-DNA construct in these mutants containing a tetramer of the cauliflower mosaic virus (CaMV) 35S enhancer, which resulted in triple functions for flanking genes dependent to the insertion locus and the genes. The mutants with the black bar indicate the T-DNA construct in these mutants has no enhancers but only double functions (knockout and promoter trapping).

L175 M0005254, M0001475 not found in TRIM database, while I was trying to search? Please describe briefly, how to search these lines in the TRIM database?
Both M0005254 and M0001475 do not have an FST, so this information is not in the FST database. When searching the TRIM phenomics database online using small grain or short panicle, M0005254 can be easily found. However, there is problem for M0001475. We started the phenomics study in 2002, and the breeders always wrote down any phenotypes they saw, without any code. I checked our database and found that some of the original written reports were not incorporated yet. We will continue to update this part. Searching Table S3 and using the “find” mode may easily recover the related information.

L211 "The phenomics data are available in Table S3 and at the TRIM website? I could not find any phenotypic data for M00052544". Pleas describe how to use TRIM to find out the phenotype of lines.
We think the reviewer meant M0005254. If you search for short panicle or small grain, M0005254 will appear. If using the second sheet of Table S3 and searching for M0005254, here are the results.
M0005254 B 2 9.5 40.5 69 102,89,87,84,82,42,31,24,21,13
where B is the subline id, 2 means the second cropping season, 9.5, 40.4, 69 mean panicle number, plant height, and heading date, respectively. The first sheet lists the id of all mutant traits, e.g. 102 is “small grain”.

L212 "how to search in Gigascience database" Please provide weblink for the same and describe briefly.
Once the paper is accepted, we will have all the information (phenomics and putative knocked-out genes) in the Gigascience database.

Reviewer #3: The present study focuses on the phenomics analysis of T-DNA tagged mutant population in rice variety Tainung 67 background.
Comments
49 "environment-friendly way" NOT "environmentally friendly" DO THE SAME ON LINE 71
Thank you very much. We used it in the revised manuscript accordingly.

49 remove "will become" replace with "becomes" DO THE SAME ON LINE 71
Thank you very much. We used it in the revised manuscript accordingly.

82 Replace Several with Two --- only two papers were cited; several refers to more than two OR cite more studies to make the word Several appropriate
Thank you very much. We did so in the revised manuscript accordingly.

84 - 89 Too long sentence. Restate into 2-3 sentences for readability.
Thank you very much. We did so in the revised manuscript accordingly.

173 - 177 What criteria was used to pick the six lines out of 30 lines found in the TRIM database that underwent sequencing?
We randomly chose these six lines in the first 50,000 accessions.

183 - What criteria were used for the 5 lines that underwent whole genome sequencing among the 90 dwarf tillering TRIM lines? Why these 5 were selected out of the 90 lines
Considering our budget limitations, we performed NGS analysis for five lines only. One of them was picked because the seeds were big (M0028590), in addition to the dwarf tillering phenotype (Ref. 19). The other four were randomly picked (Ref. 17).

For the values in table 1, are these percentage based from the 79,050 lines that have phenomics data? The total number of lines screened should be indicated as a note at the bottom of the table so that readers will have an idea.
Yes, the percentages in Table 1 are based on the 79,050 lines that have phenomics data. Yes, we added the total number at the bottom of the table in the revised manuscript.

Are the values presented in Table 2, in percentage? Please specify. Also include the total number of phenotypes screened for each mutant resource.
Yes, the values presented in Table 2 are in percentages. We added this in the current version manuscript.
