# Supplementary material for: Large-scale phenomics analysis of a T-DNA tagged mutant population
Source: Gigascience. 2017 Jul 13;6(8):1–7. doi: 10.1093/gigascience/gix055 (PMC5570018; doi:10.1093/gigascience/gix055)
Supplement: Response-to-Reviewer-Comments_Revision-1.pdf [file gix055_Response-to-Reviewer-Comments_Revision-1.pdf]

Author's Response To Reviewer Comments

We modified the version according to the editor's comments. We provided more background on the related research, we compared our resource with the 3K rice genome project, and we also added a section "Re-use potential beyond rice functional genomics".

In addition, we also sent the message back to GagiDB dataset and provided all information.
