# Supplementary material for: Large-scale phenomics analysis of a T-DNA tagged mutant population
Source: Gigascience. 2017 Jul 13;6(8):1–7. doi: 10.1093/gigascience/gix055 (PMC5570018; doi:10.1093/gigascience/gix055)
Supplement: Reviewer-2-Report-(Original-Submission).pdf [file gix055_Reviewer-2-Report-(Original-Submission).pdf]

Reviewer Report

Title: Large-scale phenomics analysis of a T-DNA tagged mutant population

Version: Original Submission **Date:** 4/4/2017

Reviewer name: Ratna Karan

Reviewer Comments to Author:

I have few questions and concerns before publication of this manuscript:

Please mention permit number for growing GM crops in the field.
L57 write the name of 3 quantitative traits.
L58 Write the name of database?
L101 please mention names of these 3 genes.
L115-116 "Our seed collection contains 86,310 lines with T1 seeds (collected from the T0 plants), and 78,757 lines with T2 seeds" Are these T2 seeds part of 86,310 lines with t1 seeds?
L116-117 "While we propagated the T2 seeds, we performed phenomics screening of T1 plants grown in the field" Were the propagation of T2 seeds and T1 phenotyping done during same growth season? How T2 seeds were selected? What was the purpose for the propagation of T2 seeds?
L150-153 "About 47,833 of the FSTs showed hits in the rice genome, which are all available in the databases NCBI GSS, RAP-db, RiceGE, or TRIM." are these all FST available in all databases?
L162-163 'The red bar indicates that the integration has triple functions (knockout, activation and promoter trapping)" How can an integration causes knockout as well as activation at the same time?
L175 M0005254, M0001475 not found in TRIM database, while i was trying to search? Please describe briefly, how to search these lines in the TRIM database?
L211 "The phenomics data are available in Table S3 and at the TRIM website? I could not find any phenotypic data for M00052544". Pleas describe how to use TRIM to find out the phenotype of lines.
L212 "how to search in Gigsscience database" Please provide weblink for the same and describe briefly.

Level of Interest

Please indicate how interesting you found the manuscript: An article of importance in its field

Quality of Written English

Please indicate the quality of language in the manuscript: Acceptable

Declaration of Competing Interests

Please complete a declaration of competing interests, considering the following questions:

- Have you in the past five years received reimbursements, fees, funding, or salary from an organisation that may in any way gain or lose financially from the publication of this manuscript, either now or in the future?
- Do you hold any stocks or shares in an organisation that may in any way gain or lose financially from the publication of this manuscript, either now or in the future?
- Do you hold or are you currently applying for any patents relating to the content of the manuscript?
- Have you received reimbursements, fees, funding, or salary from an organization that holds or has applied for patents relating to the content of the manuscript?
- Do you have any other financial competing interests?
- Do you have any non-financial competing interests in relation to this paper?

If you can answer no to all of the above, write 'I declare that I have no competing interests' below. If your reply is yes to any, please give details below.

I declare that I have no competing interests

I agree to the open peer review policy of the journal. I understand that my name will be included on my report to the authors and, if the manuscript is accepted for publication, my named report including any attachments I upload will be posted on the website along with the authors' responses. I agree for my report to be made available under an Open Access Creative Commons CC-BY license (http://creativecommons.org/licenses/by/4.0/). I understand that any comments which I do not wish to be included in my named report can be included as confidential comments to the editors, which will not be published.

I agree to the open peer review policy of the journal
