# Supplementary material for: Large-scale phenomics analysis of a T-DNA tagged mutant population
Source: Gigascience. 2017 Jul 13;6(8):1–7. doi: 10.1093/gigascience/gix055 (PMC5570018; doi:10.1093/gigascience/gix055)
Supplement: Reviewer-3-Report-(Original-Submission).pdf [file gix055_Reviewer-3-Report-(Original-Submission).pdf]

Reviewer Report

Title: Large-scale phenomics analysis of a T-DNA tagged mutant population

Version: Original Submission **Date:** 4/5/2017

Reviewer name: Roel Rabara

Reviewer Comments to Author:

The present study focuses on the phenomics analysis of T-DNA tagged mutant population in rice variety Tainung 67 background.Comments49 "environment-friendly way" NOT "environmentally friendly" DO THE SAME ON LINE 7149 remove "will become" replace with "becomes" DO THE SAME ON LINE 7182 Replace Several with Two --- only two papers were cited; several refers to more than two OR cite more studies to make the word Several appropriate84 - 89 Too long sentence. Restate into 2-3 sentences for readability.173 - 177 What criteria was used to pick the six lines out of 30 lines found in the TRIM database that underwent sequencing?183 - What criteria were used for the 5 lines that underwent whole genome sequencing among the 90 dwarf tillering TRIM lines? Why these 5 were selected out of the 90 linesFor the values in table 1, are these percentage based from the 79,050 lines that have phenomics data? The total number of lines screened should be indicated as a note at the bottom of the table so that readers will have an idea.Are the values presented in Table 2, in percentage? Please specify. Also include the total number of phenotypes screened for each mutant resource.

Level of Interest

Please indicate how interesting you found the manuscript: An article of importance in its field

Quality of Written English

Please indicate the quality of language in the manuscript: Acceptable

Declaration of Competing Interests

Please complete a declaration of competing interests, considering the following questions:

- Have you in the past five years received reimbursements, fees, funding, or salary from an organisation that may in any way gain or lose financially from the publication of this manuscript, either now or in the future?
- Do you hold any stocks or shares in an organisation that may in any way gain or lose financially from the publication of this manuscript, either now or in the future?
- Do you hold or are you currently applying for any patents relating to the content of the manuscript?
- Have you received reimbursements, fees, funding, or salary from an organization that holds or has applied for patents relating to the content of the manuscript?

- Do you have any other financial competing interests?
- Do you have any non-financial competing interests in relation to this paper?

If you can answer no to all of the above, write 'I declare that I have no competing interests' below. If your reply is yes to any, please give details below.

I declare that I have no competing interests

I agree to the open peer review policy of the journal. I understand that my name will be included on my report to the authors and, if the manuscript is accepted for publication, my named report including any attachments I upload will be posted on the website along with the authors' responses. I agree for my report to be made available under an Open Access Creative Commons CC-BY license (http://creativecommons.org/licenses/by/4.0/). I understand that any comments which I do not wish to be included in my named report can be included as confidential comments to the editors, which will not be published.

I agree to the open peer review policy of the journal
